# The research for the function evaluation of facial nerve and the mechanisms of rehabilitation training

**DOI:** 10.1097/MD.0000000000025430

**Published:** 2021-05-07

**Authors:** Han Si-Yi, Wang Ling, Yu Hai-Bo, Gou Yan-Hua, Zhong Wei-Zheng, Huang Xing-Xian, Zhang Shao-Yun, Liu Yong-Feng, Chen Yi-Rong

**Affiliations:** a Shenzhen Hospital of Traditional Chinese Medicine, Shenzhen; b Guangzhou University of Chinese Medicine, Guangzhou, China.

**Keywords:** acupuncture rehabilitation training, laser speckle blood flow imaging technology, peripheral facial paralysis, protocol

## Abstract

**Background:**

Peripheral facial paralysis (PFP) is a common peripheral neural disease. Acupuncture treatment combined with PFP rehabilitation exercises is a routine method of PFP treatment. This article is to provide a new visual and objective evaluation method for exploring the mechanism and efficacy of acupuncture treatment on PFP, and develop an interactive augmented facial nerve function rehabilitation training system with multiple training models.

**Methods:**

This prospective and observational trial will recruit 200 eligible participants for the following study. In the trial, the laser speckle contrast analysis (LASCA) technology will be applied to monitor the microcirculation of facial blood flow during acupuncture, and real-time monitoring algorithms, data sampling, and digital imaging methods will be conducted by machine learning and image segmentation. Then, a database of patient facial expressions will be built, the correlation between surface blood flow perfusion volume and facial structure symmetry will be analyzed, combined with scale assessment and electrophysiological detection. In addition, we will also explore the objectivity and effectiveness of LASCA in the evaluation of facial paralysis (FP), and the changes in blood flow microcirculation before and after acupuncture treatment will be analyzed.

**Results:**

The standard image of the facial target area with facial nerve injury will be manually segmented by the convolutional neural network method. The blood flow images of the eyelid, cheek, and mandible of the patients’ affected and healthy side will be compared and evaluated. Laser speckle blood flow symmetry Pr and its changes in FP condition evolution and prognosis outcome will be measured, and relevant characteristic signals values will be extracted. Finally, COX regression analysis method is conducted to establish a higher accuracy prediction model of FP with cross-validation based on laser speckle blood flow imaging technology.

**Conclusions:**

We use modern interdisciplinary high-tech technologies to explore the mechanism of acupuncture rehabilitation training in PFP. And we will provide evidence for the feasibility of using the LASCA technique as a typing diagnosis of FP in the acupuncture rehabilitation treatment of PFP.

**Registration number:**

ChiCTR1800019463

## 1 Introduction

Peripheral facial paralysis (PFP) is a common peripheral neural disease. The prevalence of PFP among urban residents in China is 425.7 per 100,000 population, and 258 per 100,000 individuals in rural areas, which are significantly higher than the annual incidence of 20 per 100,000 people in European and American countries, and the incidence of PFP is still increasing.^[1,2]^ Facial damage is the major clinical symptoms for PFP patients, which disfigures the appearance and have a great impact on patients’ social interaction, work and life, causing a heavy psychological burden or even derailing sufferers from society.

It is known that acupuncture treatment combined with facial paralysis (FP) rehabilitation guided by doctors is a routine method of PFP treatment in China; however, there are always 2 problems.^[3,4]^ On one hand, the acupuncture effect cannot be displayed validly in real time and it lacks convincing power in the clinical research of PFP because of the unclear acupuncture treatment mechanism, the scarcity of objective, and dynamic evaluation indicators. On the other hand, rehabilitation training has always been depended on the doctor's verbal guidance to the patient, but not the intuitive and interactive methods, so the effect of patient's FP rehabilitation training is limited after returning home.

At present, the clinical evaluations of facial nerve function after FP are mainly based on scales, of which House-Brakmann (H-B) rating scale is the most widely used. Although H-B rating scale has no specific and clear quantitative indicators to describe the severity of facial nerve injury, resulting in deficiencies in utility and reliability.^[4,5]^ In addition, the objective diagnostic indexes of PFP are neuroelectrophysiological diagnostic tests including surface electromyogram (EGM), nerve excitability measurement, facial nerve electrogram, EGM (the criterion standard for judging the prognosis and efficacy of facial nerves), blink reflex (BR, the most sensitive index for early diagnosis of FP to directly or indirectly reflect facial nerve conduction function) and F wave. Unfortunately, in clinical practice, EGM alone cannot reflect the degree of inhibition, damage and degeneration of the electrical conduction of the nerve, especially the low positive rate of detecting mild-to-moderate FP, besides, the facial nerve function cannot be evaluated in the acute stage of the lesion, making the detection lagging. BR requires professional operation, and the process is complicated, causing pain and discomfort to the patient, resulting in poor patient acceptance.^[6]^ Electrophysiological testing cannot dynamically reflect the evolution of FP but only shows the facial nerve function currently being examined. It is difficult to provide effective objective assessment of acupuncture for FP.

Facial nerve compression of FP is mainly caused by local neurotrophic vasospasm and tissue edema due to inflammation, edema, ischemia, or autonomic nervous system disorder in the facial neural canal and surrounding tissues.^[7]^ Some in-depth researches believed that facial nerve ischemia in patients with FP can lead to different degrees of blood circulation disorders, and its recovery depends on stable internal neurological environment and nutritional supply, which are based on good blood circulation.^[8,9]^ A comprehensive understanding of the blood circulation of the facial nerve can help evaluate the development and prognosis of PFP; thus, a new method for evaluating FP was proposed based on the dynamic observation of local conditions of PFP using infrared thermal imaging.^[10,11]^ Nevertheless, infrared thermal imaging has high environmental requirements, blurred imaging, poor experimental consistency, and insurmountable technical defects.^[12]^

In clinic, individualized acupuncture treatment programs are provided for FP patients according to the severity of FP, the development and change of the condition, different physical conditions, and syndromes. It is urgent to comprehensively evaluate the early stage of FP and grasp the rules of outcome and evolution of FP by noninvasive and dynamic visualization of objective evaluation indicators, which could help explore the mechanism of acupuncture treatment and make clinical acupuncture treatment for FP more predictable, targeted, and proactive.

In recent years, with the internationalization of acupuncture, more and more evidences have shown^[13,14]^ that acupuncture was scientific in treating and preventing certain diseases. Visualization research has led to qualitative changes in western medicine diagnosis and treatment, so we believe that visualization research may also become a breakthrough path for the technological advancement of Chinese medicine.^[15]^ For example, Laser Speckle Contrast Analysis (LASCA)^[16]^ can use the backward dynamic speckle contrast value generated by red blood cell movement in blood vessels to obtain blood flow velocity information and acquire a 2-dimensional high-resolution blood flow distribution image, which is a good indicator for monitoring superficial microcirculation perfusion of the skin and can be applied to PFP research. Our previous study has demonstrated that the blood flow on the affected side of the patient with FP was lower than that on the healthy side by using the technology of LASCA.^[17,18]^ Furthermore, upper and lower extremity stroke rehabilitation training system based on VR technology can be quickly copied to the FP training rehabilitation system. Here, our aim is to expolre the acupuncture mechanism on FP therapy based on LASCA technology combined with deep learning and image segmentation, which can help patients with FP recovery by developing dynamic interactive FP rehabilitation exercises depended on virtual reality technology.

## 2 Methods

### 2.1 Design

**Figure 1:**
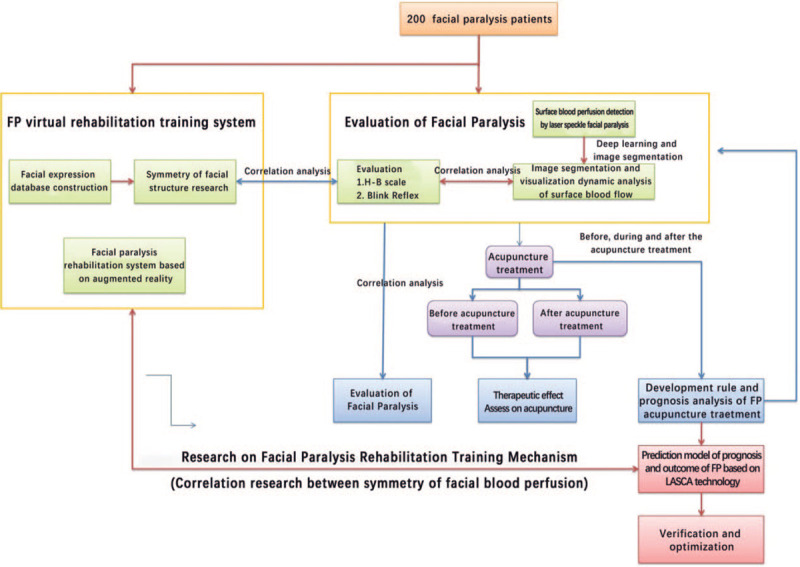
Flow chart and technical roadmap of this project.

This study is an observational trial conducted in Shenzhen Hospital of Traditional Chinese Medicine, China. After getting written informed consent, H-B scale, BR, facial structure symmetry analysis, and LASCA technique testing were performed on 200 eligible patients with PFP. The primary objective is to provide a new visual and objective evaluation method for studying the mechanism and efficacy of acupuncture treatment of FP and develop an interactive augmented reality facial nerve function rehabilitation training system with multiple training modes. The study flow chart is showed in Figure 1.

### 2.2 Sample collection

Sample size is calculated based on previous observation research results using PASS.11 software.^[18]^ Two hundred patients with FP who met the requirements were registered and their personal files were established in research database from 2020.5 to 2022.5.

### 2.3 Diagnostic criteria

Participants must meet the western medical diagnostic criteria (Table 1, with reference to the fifth edition of the textbook “Neurology” edited by Wang Weizhi), and the traditional Chinese medical (TCM) diagnostic criteria and syndrome differentiation (Tables 2 and 3, with reference to the textbook “Acupuncture and Moxibustion” edited by Shi Xuemin). The judgment of syndrome differentiation will be determined independently by 2 designated deputy physicians of TCM.

**Table 1:** Western medical diagnostic criteria for facial paralysis.

1. Medical history: urgent onset, often with a history of catching a cold or blowing, or a history of viral infection.
2. Main clinical manifestation: sudden paralysis of facial expression muscles on 1 side, disappearance of forehead lines on the sick side, nonclosable eyelids, shallowed nasolabial fold, sagging mouth angle, drum cheek, leak when whistling and food tends to be stayed between the teeth and cheek on the affected side.
3. Accompanying clinical manifestation: loss of taste of the front two-thirds of the tongue on the affected side, hyperacusis, and more tears.
4. Auxiliary examination: normal brain CT and MRI examinations

CT = computed tomography, MRI = magnetic resonance imaging.

**Table 2:** Diagnostic criteria for traditional Chinese medical of facial paralysis.

1. Sudden onset of illness, often with a history of catching a cold, mostly in spring and autumn
2. Facial stagnation, numbness and tears on one side, disappearance of forehead lines, shallowed nasolabial fold, nonclosable eyes, mouth corner pulled toward the healthy side, or pain or fever of the cheek, the ear, or behind the ear on one side.
3. Failed to close eye, drum cheek, or show teeth on 1 side
4. Abnormal EGM

EGM = electromyogram.

**Table 3:** Diagnostic criteria for TCM syndrome differentiation of facial paralysis.

1. Syndrome of wind-cold invading collaterals: sudden appearance of skewed mouth and eyes, incompletely closed eyelids, history of catching cold on the face, pale tongue, thin and white fur, floating and tight pulse.
2. Syndrome of wind-heat invading collaterals: sudden appearance of skewed mouth and eyes, incompletely closed eyelids, secondary to cold and fever, or a history of throat infections, red tongue, yellow and greasy fur, floating and rapid pulse.
3. Syndrome of wind-phlegm obstructing collaterals: sudden appearance of skewed mouth and eyes, incompletely closed eyelids, or facial jerking, numb and swollen face, with heavy head, chest tightness, or spitting, plump tongue, white and greasy fur, stringy and slippery pulse.
4. Syndrome of qi deficiency and blood stasis collaterals: more common in patients with a recovery period or a longer course of disease, tired and weak limbs, pale complexion, dizziness, skewed mouth and eyes, incompletely closed eyelids for a long time, jerking facial muscles, pale and purplish tongue, thin and white fur, thready and hesitant pulse or thready and weak pulse.

TCM = Traditional Chinese Medicine.

### 2.4 Eligibility criteria and exclusion criteria

The included patients are in accordance with the following eligibility criteria: participants meet the diagnostic criteria for PFP; participants have their first onset and the course of disease is within 3 months; participants have unilateral facial muscle paralysis; participants are aged between 15 and 70 years; participants offer informed consent and volunteered to participate in the trial.

Participants meet with these exclusion criteria and are excluded: FP of patient is secondary to other diseases, such as Gillan-Barre syndrome, otogenic facial neuritis (such as otitis media, labyrinthitis, mastoiditis, mumps, and so on), posterior cranial fossa tumors, and meningitis; patients with severe primary diseases such as diabetes, cardiovascular and cerebrovascular disease, liver disease, kidney disease, and patients with mental illness; patients with Hunt syndrome; patients who are younger than 15 years or older than 70 years, women that are pregnant or in lactating.

### 2.5 Acupuncture treatment protocol

All the patients receive acupuncture treatments 5 times/week for a total of 20 sessions over 4 weeks. In the acupuncture treatment, 14 main acupoints are used (acupoints of Yangbai, Zanzhu, Sizhukong, Sibai, Xiaguan, Yingxiang, Dicang, Jiache, Qianzheng, Yifeng on the affected side, and acupoints of Hegu, Taichong on both sides). Minor points can be added and subtracted according to different syndrome differentiation, physique and symptoms, for example, Lianquan is added when taste sense of the front two-thirds of the tongue is loss.

In addition, there is difference in the operation of acupuncture. Acupuncture operation in the acute phase should be light and shallow, without twisting and lifting, and electroacupuncture is forbidden, whereas mild-reinforcing and mild-reducing method is usually used in acupuncture of the recovery period. Electroacupuncture can be chosen for patients with facial muscle atrophy, two pairs of points including Xiaguan-Juliao and Jiache-Dicang are generally chosen and the time is last for 30 minutes. During the period of electroacupuncture, continuous waves are used, with a frequency of 1 to 2 Hz and the output intensity is based on the slight contraction of facial muscles.

### 2.6 Surface blood flow signal collection of face

PeriCam PSI laser speckle imager (Perimed, Sweden) is applied for surface blood flow signal collection of face. The parameter is set as followings: high resolution, 10 images/s of frequency, the acquisition frequency is 50 Hz, and the sampling density is medium. After that, facial blood perfusion volume is recorded over 3 periods: 5 minutes that the patient lies flat and quietly; the whole process of acupuncture; 5 minutes after needle removal. The evaluation criteria for FP in LASCA are shown in Table 4. When monitoring, the patient is in a supine position, facing directly above, and the laser emission and monitoring head are placed about 25 to 30 cm directly above the patient's face.

**Table 4:** Evaluation criteria for facial paralysis in LASCA.

1. ROI: In machine vision and image processing, machine learning and image segmentation methods are used to automatically divide the facial blood flow image into 6 regions: left and right eye area, left and right cheek area, and left and right jaw area.
2. TOI: In machine vision and image processing, periods of interest are selected. Generally, 3 periods are taken: before acupuncture (5 min), during acupuncture (20 min), after acupuncture (5 min).
3. Pr: Pr = P1 (average blood perfusion volume on the healthy side)/P2 (average blood perfusion volume on the affected side). The closer Pr is to 1, the faster the recovery, the better the curative effect, and the better the prognosis.

LASCA = laser speckle contrast analysis.

### 2.7 Nerve electromyogram signal collection using H-B rating scale

Viking Quest EMG system (Thermo Nicolet Corporation, America) is utilized for nerve electromyogram (EGM) signal collection, and room temperature is maintained at 18°C to 25°C. When the patient is performed with BR of facial nerve, we choose a repetitive waveform to measure the shortest latency of R1 and R2 (Table 5). After that facial nerve function before and after acupuncture intervention is assessed by using H-B rating scale (Table 6).

**Table 5:** Evaluation criteria for facial paralysis in BR.

1. The latency of R1 is 10.0 ± 0.6 ms, when the latency of R1 is ≥2 ms and the difference between the latency of bilateral R1 is ≥2.0 ms, these data can be judged as abnormal.
2. The latency of R2 is 29.3 ± 1.8 ms, and the latency that ≥34 ms can be judged as abnormal.
3. Undetectable or absent signal of R1, R2, and contralateral R2 ′is considered as abnormal.

BR = blink reflex.

**Table 6:** H-B rating scale.

1. Mild: H-B rating scale has a local score of II
2. Moderate: H-B rating scale has a local score of III-IV
3. Moderate to severe: H-B rating scale has a local score of V
4. Severe: H-B rating scale has a local score of VI

H-B = House-Brakmann.

### 2.8 Adverse events and other unintended effects

We will collect, assess, report, and manage solicited and spontaneously reported adverse events and other unintended effects of trial interventions or trial conduct, with which we could evaluate the safety of acupuncture treatment.

### 2.9 Statistical analysis

SPSS 20.0 statistical software is used for statistical analysis of data (SPSS Inc, Chicago, IL). The correlation between H-B score, BR, and Pr would be analyzed by correlation analysis, the closer the correlation coefficient is to 1 or −1, the stronger the correlation, the closer the correlation coefficient to 0, the weaker the correlation. Radit analysis is adopted to analyze H-B facial nerve function rating scale. In terms of surface blood perfusion signal analysis, statistical analysis is mainly performed on Pr values before and after treatment. Data are expressed as “mean ± SD” and homologous paired sample t test is utilized. COX regression analysis is used to analyze the development of FP and the prediction on model of its prognosis and outcomes. *P* < .05 indicates that the difference is statistically significant, as well as *P* < .01 means that the statistical significance is remarkable.

## 3 Results

### 3.1 Research on automatic extraction and visual analysis method of interested region based on deep learning

Based on the image segmentation framework of convolutional neural networks (CNN, one of the most commonly used deep learning models), a local image block centered on a pixel is taken as a processing object, and a method for segmenting a facial image of a patient with FP is implemented by pixel-by-pixel classification. The basic steps are as follows. Above all, the experts manually segment the region of interest on the face image to obtain the segmented image of gold standard and initially identify 100 images. Subsequently, the experts extract training samples, which are the local image block in face images. Researchers select some pixels from the region of interest, and use these pixels as the center to extract local image blocks as positive samples, the same, extract local image blocks from other regions as negative samples. Training sample set is represented as $\text{X}=\left\{\left({\text{x}}_{1},y_{1}\right)|\text{i}=\text{1,2,}\cdots \text{m}\right\}$, ${\text{X}}_{1}\in {\text{R}}^{\omega \times \omega \times \text{h}}$(ω×ω: the size of local image block, h=3: the number of image channels) stands for the local image block, ${\text{y}}_{\text{i}}\in \{0,1\}$ (1: interested region, 0: noninterested region) means image block marking. Thirdly, rotating, translating, shearing, and symmetrical flipping are performed on local image block samples to enlarge the sample capacity and prevent the overfitting of CNN to a certain extent, and then CNN network including convolutional layer, pooling layer, fully connected layer, and Softmax classification loss layer is set up. During the process of building CNN network, a linear correction unit ReLU activation function (f(x)=max(0,x)) is added after the combination of each convolutional layer and pooling layer, improving the training speed and accuracy, and the Softmax classification loss layer defines the objective function of the CNN network $1(\text{X,C})=-\frac{\log{\text{e}}^{{\text{X}}_{\text{C}}}}{\sum_{\text{k}=1}^{\text{C}}{\text{e}}^{\text{Xk}}}$ (x: training data, c: data category). At last, the CNN network is trained using back propagation algorithm (BP). Combining all the above steps, the overall operation process is that, when a new test image is input, with each pixel of the test image as the center, the extracted local image blocks are input into the trained CNN network, the output is the probability value that the central pixel belongs to each category, and the pixels are classified according to the magnitude of the probability value.

### 3.2 Establishment of predictive model for prognosis of FP based on LASCA technology and research on rehabilitation training mechanism

The researchers automatically segregate 3 areas of the eyelid, cheek, and jaw of the affected and healthy side of the patient's facial blood flow image are by machine learning methods and synchronize them. At the same time, the common FP assessment method is incorporated into a unified mathematical statistical model, and the law of change in LASCA symmetry value Pr and the development, evolution, and prognosis of FP is observed. The relevant characteristic signals are extracted, and a COX regression analysis method was adopted to establish a highly accurate prediction model for the prognosis of FP based on LASCA technology, and cross-validation is performed.

### 3.3 Research on 3D facial structure symmetry

The main work is to study the facial 3D motion analysis system composed of vision measurement subsystem, data processing and result output subsystem, and support subsystem and the principle of system is multi-eye stereo vision measurement principle. The working process is as followed. The visual measurement subsystem measures the reflective marking points pasted on the human face, the camera captures the reflected light from the face, and the multichannel synchronization controller ensures that each capture of camera is highly synchronized, then the data processing and result output subsystem performs coordinate conversion according to the calibration results, compares changes in the spatial position of the marker points between different frames of images, and calculates the motion parameters of the marker points. During the measurement, the indexes of the healthy and affected sides of patients with unilateral FP are measured separately, and the symmetry of the structure of the affected side and the healthy side is studied.

### 3.4 Research on facial nerve function training system based on augmented reality technology

The authors will develop mobile software that runs on Android and IOS platforms. The software can photograph the face through the front camera of the phone and automatically recognize the algorithms for various parts of the face to achieve accurate recognition of each part of the face. Then the virtual training content can be displayed on the real image of the identified corresponding part, and the patient can perform rehabilitation game training according to the training content guidance to achieve effective rehabilitation training for facial nerve function while playing. Furthermore, the system will evaluate the patient's rehabilitation training effect according to the 3-dimensional grading evaluation method of facial nerve function for patients with FP and generate an evaluation report, which can be transmitted to the rehabilitation physician via the network, so that the rehabilitation physician can grasp the patient's recovery situation and guide the training in time.

## 4 Discussion

With the high-burden work and irregular diet in modern life, PFP has become a common clinical peripheral nervous system disease resulting in an increasing incidence throughout the world.^[1–3]^ Especially in Shenzhen, China, located along the coast, its incidence of PFP has been higher than other cities as its faster pace of life and its burden pressure on residents. An effective diagnosis and treatment on PFP can not only reduce the damage of patients’ appearance, the negative influence on social interaction, work and life, but also eliminate the psychological pressure, improve the quality of life, reduce or prevent the occurrence of sequelae of PFP patients.^[15–18]^

FP has always been a predominant disease with significant curative effect in the acupuncture department of Shenzhen Traditional Chinese Medicine Hospital. Acupuncture treatment combined with FP rehabilitation training serve as a routine method of FP treatment in the acupuncture department, but the mechanism of acupuncture treatment has not been fully cleared, and the lack of objective dynamic index evaluation has led to the inability to demonstrate the acupuncture effect in real-time and efficacy in FP clinical research.^[19,20]^ LASCA uses the backward dynamic speckle contrast value generated by the movement of red blood cells in blood vessels to obtain blood flow velocity information, it has the technical characteristics of visualization, nondestructive, real-time, and high precision, and it is also a good indicator for monitoring the superficial microcirculation of the skin. Thus, this project is to conduct related research on the acupuncture mechanism of FP to assist in the diagnosis and treatment of FP based on LASCA. This research can not only provide a new objective evaluation method for the mechanism of acupuncture treatment of PFP, combined with the relevant characteristics of its technical means, but it can also be quickly applied to ordinary clinics and quickly promote clinical.

## Author contributions

WL designed the study and serve as an arbiter for a final decision throughout the entire procedure. HS-y and WL perform data collected data from clinical and conducted the detection on patients. YG, WZ, and XH performed data analysis and evaluated the accuracy of the whole process. SZ and YL were responsible for contacting with the patients. HS-y, WL, and YC provided support for modifications of English writing. All authors contributed to the entire study conduction, and approved the final version submitted for publication.

**Conceptualization:** Weizheng Zhong.

**Data curation:** Siyi Han, Haibo Yu, Yanhua Guo, Weizheng Zhong, Shaoyun Zhang, Yongfeng Liu.

**Formal analysis:** Siyi Han, Weizheng Zhong, Shaoyun Zhang.

**Investigation:** Siyi Han, Ling Wang, Shaoyun Zhang, Yongfeng Liu.

**Methodology:** Siyi Han, Haibo Yu, Xingxian Huang, Shaoyun Zhang, Yongfeng Liu, Yirong Chen.

**Project administration:** Ling Wang.

**Resources:** Haibo Yu, Xingxian Huang, Yirong Chen.

**Software:** Yanhua Guo, Xingxian Huang, Yirong Chen.

**Supervision:** Ling Wang.

**Validation:** Ling Wang, Yirong Chen.

**Writing – original draft:** Haibo Yu.

**Writing – review & editing:** Ling Wang, Yanhua Guo, Yongfeng Liu.
